# A Synthetic Approach of New Trans-Substituted Hydroxylporphyrins

**DOI:** 10.1155/2010/307696

**Published:** 2010-06-10

**Authors:** Dimitra Daphnomili, Maria Grammatikopoulou, Catherine Raptopoulou, George Charalambidis, Theodore Lazarides, Athanasios G. Coutsolelos

**Affiliations:** ^1^Laboratory of Bioinorganic Coordination Chemistry, Department of Chemistry, School of Sciences, University of Crete, P.O. Box 2208, Heraklion 71003, Crete, Greece; ^2^Institute of Materials Science, NCSR “Demokritos”, Aghia Paraskevi Attikis, 15310, Greece

## Abstract

The synthesis of new *trans* A_2_B_2_-substituted porphyrins bearing oxygenic substituent (methoxy, acetoxy, hydroxy) at the periphery of the ring are described. All of the synthesized products were characterized by ^1^H-N.M.R., ^13^C-N.M.R., and H.R.M.S. Electrochemical studies revealed two one-electron oxidations and two reductions. In addition, the X-ray structure of one methoxy-derivative was determined.

## 1. Introduction

In the last years porphyrin derivatives have been developed or are under development for use as photosensitizers for photoelecronic materials such as sensors [1] and photosensitized solar cells [2]. Because of their interesting optical properties, porphyrin molecules have been investigated as artificial light harvesting antennae. Carbon-based donor-acceptor hybrid materials have been reported where, in many cases, the porphyrin molecule is covalently attached [3, 4]. Among the great diversity of porphyrins with a specific pattern of substituents, *trans*-substituted porphyrins with functional groups at the periphery of the ring act as precursors for supermolecular structures.

During the past decades a great effort has been directed towards the synthesis of porphyrins [5, 6]. Porphyrins with nearly all sorts of substituents at the periphery of the 18*π* *-*electron system are now accessible. The synthetic procedures followed were mainly based on the Adler-Longo reaction of the condensation of pyrrole with various aldehydes.

In the field of *trans*-substituted porphyrins an attractive route for the synthesis of these key structural components found in a wide range of model systems [7] was developed by Lindsey's group [8–10]. The synthetic approach of Lindsey's group was based on the convenient preparation of 5-substituted dipyrromethanes [8]. Condensation of a dipyrromethane with an aldehyde in a MacDonald-type synthesis has been used for the preparation of a wide range of *trans* A_2_B_2_ type *meso*-substituted porphyrins [8, 11, 12].

 Based on this method we tried to explore the possibility of the synthesis of *meso*-substituted *trans* hydroxyporphyrins due to the ability of the hydroxy group to link substructures over the porphyrin plane. Hydroxyporphyrins can act as precursors for the synthesis of porphyrin dimers serving as host molecules [13]. Furthermore a series of hydroxyporphyrins has been tested as photosensitizers in photodynamic therapy (PDT) [14, 15]. For their synthesis the methoxy- or acetoxy-derivatives were prepared first.

## 2. Experimental

### 2.1. Measurements

^1^H-N.M.R. and ^13^C-N.M.R. spectra were recorded on a Bruker AMX-500 MHz N.M.R. spectrometer using chloroform-D_3_ as a solvent. Resonances in the ^1^H-N.M.R were referenced versus the residual proton signal of the solvent.

Absorption spectra were collected on a Perkin-Elmer Lamda 6 grating spectrophotometer. Cyclic voltammetry experiments were performed in an AUTOLAB PGSTAT20. MS spectra were recorded on Bruker MALDI TOF/TOF ultraflextreme.

X-ray diffraction measurements were conducted on a STOE IPDS II diffractometer using graphite-monochromated Mo K_*α*_ radiation. A dark blue crystal with approximate dimensions 0.50 × 0.40 × 0.14 mm was mounted on a capillary. Intensity data were recorded using 2*θ* scan (2*θ*_max _= 46.5, 1°/min). The structure was solved by direct methods and refined on *F*_*o*_^2^ values using **SHELX** [16]. All nonhydrogen atoms were refined anisotropically; all of the hydrogen atoms were introduced at calculated positions as riding on bonded atoms and were refined isotropically.

### 2.2. Synthesis of Porphyrinic Compounds

The preparation of 5-mesityl dipyrromethane was based on previously published procedures [8].

#### 2.2.1. 5.15 Dimesityl-10,20 Bis(3-Methoxyphenyl)Porphyrin **1**

3.8 mmol (1 gr) of 5-mesityl dipyrromethane and 3.8 mmol of 3-methoxybenzaldehyde were dissolved in 400 mL of CH_2_Cl_2_ (A.C.S. grade) under argon atmosphere. 7.12 mmol TFA were added and the reaction mixture was stirred for 30 min at room temperature. 3.8 mmol (0.86 gr) of DDQ were added and the mixture was further stirred for 1 hour. The reaction mixture was filtered through a column of Al_2_O_3_ (6 cm × 8 cm) using CH_2_Cl_2_ as eluent until the color of the solution was pale brown. The solvent was removed under reduced pressure and the solid was dissolved in 50 mL of toluene, heating at reflux for 1 hour, after the addition of 0.38 mmol of DDQ. After cooling at room temperature the solvent was removed and the solid was purified by a column chromatography. A column of Al_2_O_3_ was performed with CH_2_Cl_2_ as eluent (yield 22%):

MS: [M]^+^ 758.3631,
UV-Visible: *λ*_max _(toluene, 5.3 × 10^−5^ M) /(log *ε*/M^−1^ cm^−1^): 401 (sh, 4.79), 419 (Soret, 5.54), 482 (sh, 3.84), 513 (Q, 4.28). 550 (Q, 3.90), 591 (Q, 3.92), 647 (Q, 3.72),
^1^H-N.M.R. (500 MHz, CDCl_3_, 300 K) *δ* = 8.86 (d, 4H, *J* = 4.6 Hz, pyrrole); 8,71 (d, 4H, *J* = 4.6 Hz, pyrrole). *Phenyl Group*: *δ* = 7.84 (m, 4H, 2,6-ph); 7.66 (tr, 2H, *J* = 8 Hz, 3-ph); 7.35 (dd, 2H, *J* = 8 Hz, 4-ph); 4.02 (s, 6H, −OCH_3_). *Mesityl Group*: *δ* = 7.31 (s, 4H, 3,5-mes); 1.88 (s, 12H, 2,6-mes); 2.66 (s, 6H, 4-mes); −2.60 (s, 2H, N-pyrrole).

#### 2.2.2. 5.15 Dimesityl-10,20 Bis(2-Methoxyphenyl)Porphyrin **2**

The standard procedure described above was followed obtaining 0.38 gr of **2** as a mixture of the two atropisomers (yield 26%):

MS: [M]^+^ 758.3630,
UV-Visible: *λ*_max _(toluene, 6.2 × 10^−5^ M)/(log *ε* /M^−1^ cm^−1^): 401 (sh, 4.78), 419 (Soret, 5.55), 482 (sh, 3.87), 515 (Q, 4.30), 550 (Q, 3.91), 591 (Q, 3.91), and 647 (Q, 3.76),
^1^H-N.M.R. (500 MHz, CDCl_3_, 300 K) *δ* = 8.73 (d, 4H, *J* = 4.6 Hz, pyrrole); 8.65 (d, 4H, *J* = 4.6 Hz, pyrrole). *Phenyl Group*: *δ* = 8.03 (d, 2H, *J* = 7.2 Hz, 6-ph); 7.82 (tr, 2H, *J* = 7.6 Hz, 4-ph); 7.36 (dd, 4H, *J* = 8.5 Hz, 3,5-ph); 3.63 (s, 6H, −OCH_3_). *Mesityl Group*: *δ* = 7.29 (s, 4H, 3,5-mes); 1.87 (s, 12H, 2,6-mes); *^(a)^*1.89 (s, 6H); *^(b)^*1.86 (s, 6H); *^(b)^*2.65 (s, 6H, 4-mes); −2.51 (s, 2H, N-pyrrole).
(*a*) *α*, *β* atropisomer,
(*b*) *α*, *α* atropisomer.

#### 2.2.3. 5.15 Dimesityl-10,20 Bis(4-Acetoxyphenyl)Porphyrin **3**

The procedure described for **1** was followed. The product was obtained after repeat washings with cold ethanol and recrystallization from CH_2_Cl_2_/Hexane/EtOH (10/1/5 v/v/v) at −5°C overnight (yield 27%):

UV-Visible: *λ*_max _(toluene, 1.6 × 10^−4^ M)/(log *ε* /M^−1^ cm^−1^): 399 (sh, 4.85), 418 (Soret, 5.48), 480 (sh, 3.98), 513 (Q, 4.36), 549 (Q, 4.04), 591 (Q, 4.02), and 647 (Q, 3.92),
^1^H-N.M.R. (500 MHz, CDCl_3_, 300 K) *δ* = 8.85 (d, 4H, *J* = 4.5 Hz, pyrrole); 8.73 (d, 4H, *J* = 4.5 Hz, pyrrole). *Phenyl Group*: *δ* = 8.25 (d, 4H, *J* = 8 Hz, 2,6-ph); 7.52 (d, 4H, *J* = 8.5 Hz, 3,5-ph); 2.52 (s, 6H, −OOCCH_3_). *Mesityl Group*: *δ* = 7.32 (s, 4H, 3,5-mes); 2.67 (s, 6H, 4-mes); 1.89 (s, 12H, 2,6-mes); −2.51 (s, 2H, N-pyrrole).

#### 2.2.4. 5.15 Dimesityl-10,20 Bis(4-Methoxyphenyl)Porphyrin **4**

The standard procedure described above was followed (yield 26%):

UV-Visible (CH_2_Cl_2_): *λ*_max _(toluene, 2 × 10^−4^ M)/(log *ε*/M^−1^ cm^−1^): 420 (5.67), 516 (4.27), 552 (3.95), 592 (3.75), and 649 (3.71),
^1^H-N.M.R. (500 MHz, CDCl_3_, 300 K) *δ* = 8.85 (d, 4H *J* = 5 Hz, pyrrole); 8.71 (d, 4H, *J* = 4.5 Hz, pyrrole). *Phenyl Group* *δ* = 8.16 (d, 4H, *J* = 7.5 Hz, 2,6-ph); *δ* = 7.30 (m, 4H, 3-ph); *δ* = 4.10 (s, 6H, −OCH_3_). *Mesityl Group*: *δ* = 7.30 (s, 4H, 3,5-mes); 2.65 (s, 6H, 4-mes); 1.87 (s, 12H, 2,6-mes); −2.56 (s, 2H, N-pyrrole).

#### 2.2.5. 5.15 Dimesityl-10,20 Bis(3-Hydroxyphenyl)Porphyrin **5**

0.079 mmol (0.06 gr) of porphyrin **1** was dissolved in 8 mL of dry CH_2_Cl_2_ under Ar atmosphere. The solution was cooled at −78°C and BBr_3_ (1.85 mmol) was added dropwise under vigorous stirring. The reaction mixture was allowed to stand at r.t. for 5 hours. Aqueous saturate NaHCO_3_ was added carefully and the organic layer was washed with saturate NaCl solution and dried over MgSO_4_. After the removal of the solvent the product was chromatographied on SiO_2_ column (2 cm × 4 cm). With CH_2_Cl_2_/EtOH (100/0.2 v/v), traces of unreacted porphyrin were eluted while the product was obtained with CH_2_Cl_2_/EtOH (100/5 v/v) as eluents (yield 85%):

MS: [M+H]^+^ 731.3399,
UV-Visible: *λ*_max _(toluene, 4.2 × 10^−5^ M)/(log *ε* /M^−1^ cm^−1^): 402 (sh, 4.80), 420 (Soret, 5.54), 480 (sh, 3.98), 515 (Q, 4.29), 552 (Q, 4.06), 593 (Q, 3.95), and 650 (Q, 3.89),
^1^H-N.M.R. (500 MHz, CDCl_3_, 300 K) *δ* = 8.86 (d, 4H, *J* = 4.5 Hz, pyrrole); 8.71 (d, 4H, *J* = 4.5 Hz, pyrrole). *Phenyl Group*: *δ* = 7.82 (d, 2H, *J* = 7.8 Hz, 6-ph); 7.70 (s, 2H, 2-ph); 7.60 (tr, 2H, *J* = 8 Hz, 5-ph); 7.25 (d, 2H, *J* = 7 Hz, 4-ph); 5.45 (s br, 2H, OH).* Mesityl Group*: *δ* = 7.26 (s, 4H, 3,5-mes); 2.66 (s, 6H, 4-mes); 1.79 (s, 12H, 2,6-mes); −2.60 (s, 2H, N-pyrrole).

#### 2.2.6. 5.15 Dimesityl-10,20 Bis(2-Hydroxyphenyl)Porphyrin **6**

The procedure was the same as for compound **5**. Compound **6** is a mixture of two atropisomers that were separated by column chromatography on SiO_2_ (5 cm × 2 cm). The*α*, *β*(R_f_: 0.95 in CH_2_Cl_2_) is eluted with CH_2_Cl_2_/Hexane (6/4 v/v) and *α*, *α*(R_f_: 0.25 in CH_2_Cl_2_) is eluted with 0.5% EtOH /CH_2_Cl_2_.

MS: [M+H]^+^ 731.3398,
UV-Visible: *λ*_max _(toluene, 3.4 × 10^−4^ M)/(log *ε* /M^−1^ cm^−1^): 402 (sh, 4.78), 420 (Soret, 5.55), 480 (sh, 3.83), 515 (Q, 4.32), 552 (Q, 3.99), 593 (Q, 3.92), and 650 (Q, 3.86),
6**α** **β**: ^1^H-N.M.R. (500 MHz, CDCl_3_, 300 K) *δ* = 8.84 (d, 4H, *J* = 4.5 Hz, pyrrole); 8.74 (d, 4H, *J* = 4.5 Hz, pyrrole). *Phenyl Group*: *δ* = 8.0 (dd, 2H, *J* = 7 Hz, 6-ph); 7.73 (tr, 2H, *J* = 8 Hz, 4-ph); 7.37 (d, 2H, *J* = 8.5 Hz, 5-ph); 7.34 (d, 2H, *J* = 8 Hz, 3-ph); 5.37 (s br, 2H, −OH).* Mesityl Group*: *δ* = 7.31 (s, 4H, 3,5-mes); 2.65 (s, 6H, 4-mes); 1.85 (s, 12H, 2,6-mes); −2.59 (s, 2H, N-pyrrole),
6**α** **α**: ^1^H-N.M.R. (500 MHz, CDCl_3_, 300 K) *δ* = 8.85 (d, 4H, *J* = 4.5 Hz, pyrrole); 8.74 (d, 4H, *J* = 4.5 Hz, pyrrole). *Phenyl Group*: *δ* = 8.03 (dd, 2H, *J* = 7.5 Hz, 6-ph); 7.71 (tr, 2H, *J* = 7.5 Hz, 4-ph); 7.37 (d, 2H, *J* = 8 Hz, 5-ph); 7.34 (d, 2H, *J* = 8 Hz, 3-ph); 5.32 (s br, 2H, −OH).* Mesityl Group*: *δ* = 7.30 (s, 4H, 3,5-mes); 2.65 (s, 6H, 4-mes); 1.88 (s, 6H); 1.83 (s, 6H); −2.58 (s, 2H, N-pyrrole).

#### 2.2.7. 5.15 Dimesityl-10,20 Bis(4-Hydroxyphenyl)Porphyrin **7**

**Method 1**

The procedure was the same as for compound **5** and compound **4**.

**Method 2**

0.25 mmol (0.2 gr) of porphyrin **3** were added in 10 mL of THF. 7.38 mmol KOH were dissolved in 5 mL of EtOH and the resulting alcoholic solution was added dropwise. The solution was stirred for 30 min at room temperature and then refluxed for a further 2 hours. After cooling at room temperature the solution was acidified by carefully adding glacial acetic acid. 15 mL of CH_2_Cl_2_ were added and the organic layer was washed with sat. NaCl solution. After being dried over MgSO_4_, the solvent was removed giving 0.165 gr of 7 (yield 90%):

MS: [M+H]^+^ 731.3399,
UV-Visible: *λ*_max _(toluene, 6.2 × 10^−5^ M)/(log *ε* /M^−1^ cm^−1^): 402 (sh, 4.60), 420 (Soret, 5.30), 478 (sh, 3.79), 515 (Q, 4.04), 550 (Q, 3.84), 592 (Q, 3.76), and 650 (Q, 3.71),
^1^H-N.M.R. (500 MHz, CDCl_3_, 300 K) *δ* = 8.85 (d, 4H, *J* = 4.5 Hz, pyrrole); 8.67 (d, 4H, *J* = 4.5 Hz, pyrrole). *Phenyl Group*: *δ* = 8.03 (d, 4H, *J* = 6.5 Hz, 2,6-ph); 7.19 (d, 4H, *J* = 6 Hz, 3,5-ph). *Mesityl Group*: *δ* = 7.38 (s, 4H, 3,5-mes); 2.61 (s, 6H, 4-mes); 1.82 (s, 12H, 2,6-mes); −2.60 (s, 2H, N-pyrrole).

## 3. Results and Discussion

**Scheme 1:**
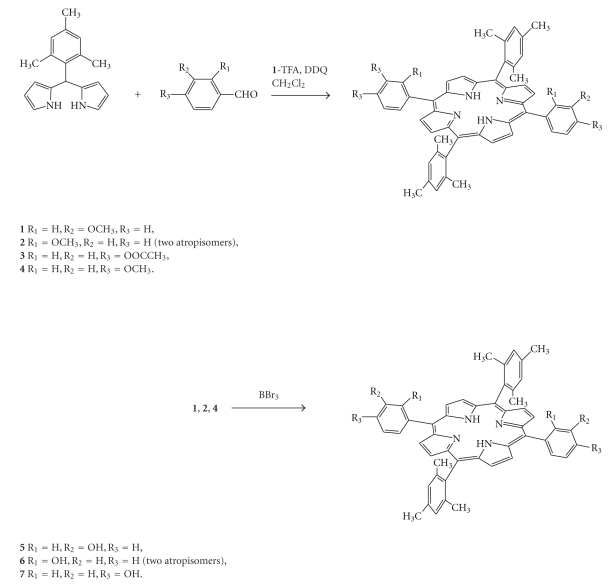
Reaction scheme.

Following Lindsey's methodology, *trans*-methoxyporphyrins **1**, **2 **and **4 **were synthesized as precursors for **5 **and **6 **while for compound **7 ** the precursors were **3** and **4 **(Scheme 1). 

The choice of acetoxy- or methoxy- as protecting groups was based on published results for the formation of a dipyrrole product from an attempted synthesis of arylporphyrins with *o*-acetoxybenzaldehyde [17]. 

**Scheme 2:**
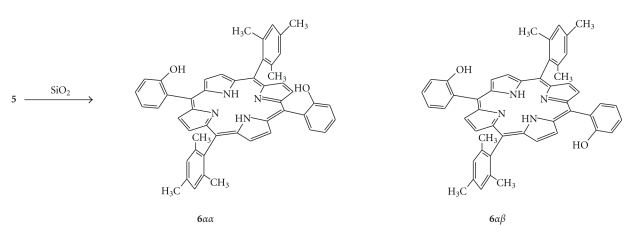
6***αα*** and 6***αβ*** atropoisomers.

Compound **2** is a mixture of atropisomers that proved to be inseparable despite our repeated efforts for chromatographic separation. Compounds **5** and **6 **were obtained by cleavage of the methyl ether by BBr_3_ (Scheme 1), while **7** is obtained by alkaline hydrolysis of the ester group or alternative by cleavage of the methoxy group. The two isomers of compound **6 **(Scheme 2) in contrast to these of **2** are easily separated by silica gel chromatography. 6**α** **β** is eluted with CH_2_Cl_2_/Hexane (6/4 v/v) while the more polar 6**α** **α** is eluted with 0.5% EtOH /CH_2_Cl_2_.

The two isomers (Scheme 2 **)** were characterized by ^1^H-N.M.R. spectroscopy. A characteristic feature is that in 6**α** **β**the *o*-Me of the mesityl group appears as a singlet while in 6**α** **α** the *o*-Me group gives two separate singlets, while no other remarkable spectroscopic difference was observed for the two isomers. In **2** since it is a mixture of the two isomers its N.M.R. spectrum shows these three groups of peaks. For derivates **3 **and **1** the *o-*H and *m-*H are equivalent giving one signal for each group. The hydrolysis product **5** the *o*-Hare no longer equivalent resonating at 7.82 ppm and 7.70 ppm.

 Characteristic in the ^13^C-N.M.R. is the signal at 170 ppm for the carbonyl carbon of **3** and at 56 ppm of −OCH_3_ group for **1 **and **2** that disappears in the ^13^C-N.M.R. spectra of the hydrolysis products. Similar characteristic I.R. peaks for **3 **at 1763 cm^−1^ for *ν*(C=O) str. no longer exist in **7** while they are also observed two new peaks, one at 1162 cm^−1^ and another one at 1200 cm^−1^ for (C–O) stretching vibrations. In methoxy derivates two bands, one at 1050 cm^−1^ [**ν**(C–O–C) sym. str.] and one at 1282 cm^−1^ [**ν**(C–O–C) asym. str.], are observed.

 For all of the methoxy derivatives electrochemical studies were performed by cyclic voltammetry. The redox potentials measured are the typical ones for meso-substituted porphyrins [18] that exhibited two one-electron reversible oxidations and two one-electron reversible reductions (Table 1).

**Figure 1:**
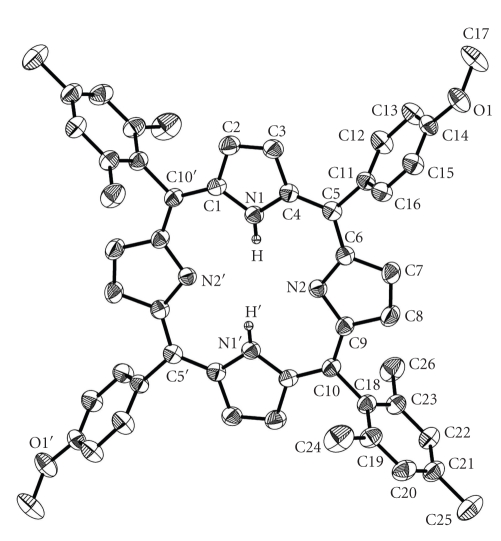
Partially labeled plot of **4** with ellipsoids drawn at 30% thermal probability. Hydrogen atoms have been omitted for clarity. Primed atoms are generated by symmetry operation: (′)-*x*, -*y*, -*z*.

 The structure of derivative **4** is centrosymmetric (Table 2) and the asymmetric unit contains half of the porphyrin molecule and one water solvate molecule, which was found disordered and refined over three positions with occupation factors summing one (Figure 1). 

The rather large values of dihedral angles formed between the porphyrin C_20_N_4_ mean plane, the mesityl phenyl ring (84.72°), and the methoxyphenyl ring (65.12°) indicate that there is no twist distortion of the porphyrin skeleton, together with the small average absolute displacement of the C_m_ atom (0.032 Å) from the poprhyrin core. The displacement of the two −OCH_3_  groups is 0.643 Å alternative from the porphyrin plane.

In conclusion in this work we have reported the preparation of new porphyrinic complexes bearing the appropriate groups in order to functionalize specific sides of the aromatic macrocycle. The formed complexes are fully characterized. The formation and the properties of macromolecule structures with the formed complexes as precursors will be published elsewhere.

## Figures and Tables

**Table 1:** Redox data of dimethoxy derivatives^(a)^.

Compound	E_1/2ox_ (V versus SCE)	E_1/2red_ (V versus SCE)
Compound **1**	0.96; 1.40	−1.34; −1.68
Compound **2**	0.94; 1.36	−1.34; −1.71
Compound **4**	0.92; 1.37	−1.33; −1.66

^(a)^Redox potentials were determined by cyclic voltammetry at room temperature in dry and deoxygenatrd CH_2_Cl_2_ containing 0.1 M of tetrabutylammonium hexafluorophosphate as supporting electrolyte and a solute concentration in the range of 1.5 × 10^−3^ M. A Saturated Calomel Electrode (SCE) was used as reference. Under these conditions, the reversible oxidation of ferrocene was E_1/2*F*_*c*__ = +0.47 V. The error on the reported potentials is ±0.01 V.

**Table 2:** Crystallographic data for **4**  2H_2_O.

	**4** 2H_2_O
Formula	C_52_H_50_N_4_O_4_
*Fw*	794.96
Space group	*P*2_1_/*n *
*a *(Å)	17.288(4)
*b *(Å)	8.2587(17)
*c* (Å)	17.829(4)
*α* (°)	90
*β* (°)	106.15(3)
*γ* (°)	90
*V* (Å^3^)	2445.1(9)
*Z*	2
*T* (°C)	25
Radiation	Mo K*α*
*ϱ*_calcd_ (g cm^−3^)	1.080
*μ* (mm^−1^)	0.069
Reflections with *I* > 2*σ*(*I*)	2338
*R*_1_^a^	0.0723
*w* *R*_2_^a^	0.1890

^a^ *w* = 1/[*σ* ^2^(*F*_*o*_^2^) + (*α* *P*)^2^ + *b* *P*]  and  *P* = [max  (*F*_*o*_^2^, 0) + 2*F*_*c*_^2^]/3.
